# Case Report: Reduction in post-amputation phantom limb pain intensity accompanying the onset of phantom limb telescoping

**DOI:** 10.3389/fpain.2024.1409352

**Published:** 2024-10-09

**Authors:** Andrea Aternali, Heather Lumsden-Ruegg, Lora Appel, Sander L. Hitzig, Amanda L. Mayo, Joel Katz

**Affiliations:** ^1^ Department of Psychology, York University, Toronto, ON, Canada; ^2^ School of Health Policy & Management, York University, Toronto, ON, Canada; ^3^ St. John’s Rehab Research Program, Sunnybrook Research Institute, Sunnybrook Health Sciences Centre, Toronto, ON, Canada; ^4^ Physical Medicine & Rehabilitation, Temerty Department of Medicine, University of Toronto, Toronto, ON, Canada

**Keywords:** case report, phantom limb pain, residual limb pain, post-amputation pain, telescoping, phantom sensations

## Abstract

**Introduction:**

Individuals with limb loss frequently report post-amputation phenomena, including nonpainful phantom sensations, phantom limb pain (PLP), and residual limb pain (RLP). Although post-amputation pain is common, not all patients benefit from widely accepted treatments. A greater understanding of phantom limb “telescoping”, the experience of one's phantom hand or foot gradually approaching the residual limb, may assist in developing more effective interventions for reducing post-amputation pain. This case report explores the relationships between PLP, RLP, telescoping, and psychosocial experience in one person with a lower limb amputation. The aim of this case is to illustrate one possible relationship between telescoping and PLP as the mechanisms linking the two remain equivocal.

**Methods:**

The participant is a 35-year-old male who underwent a transfemoral amputation due to a traumatic injury to his right leg approximately 4 years prior. He responded to questionnaires evaluating demographic and health-related information (e.g., age, sex, marital status, reason for amputation), pain and psychological variables via the Brief Pain Inventory (BPI-SF), ID Pain Questionnaire (IDPQ), Pain Catastrophizing Scale (PSC-4), Patient Health Questionnaire-4 (PHQ-4), Life Orientation Test-Revised (LOT-R), Connor-Davidson Resilience Scale (CD-RISC2), and Chronic Pain Acceptance Questionnaire (CPAQ-8) and telescoping, measured by a newly developed app. The participant completed a semi-structured interview that was designed to ascertain patterns in the overlapping experience of phantom limb telescoping and post-amputation pain.

**Results:**

The participant rated his average PLP as 10 on a Numeric Rating Scale (NRS) from 0 (“no pain”) to 10 (“worst pain imaginable”) shortly after amputation. Approximately 12 months later, the participant noticed a shortening of his phantom limb, with a concurrent decrease in PLP. At present, his average NRS pain intensity is a 5/10. The participant described how the daily, debilitating PLP intensity diminished to weekly, manageable pain over time. Most notably, his responses on questionnaires were consistent with neuropathic PLP, mild to moderate levels of pain interference, a high level of catastrophic thinking about pain, low optimism, and mild symptoms of anxiety and depression.

**Discussion:**

In this report, telescoping appeared to be preceded by an initial reduction in PLP intensity but these findings are based on a single case report and must be replicated with a large sample size before we have a clearer idea of the relationship between telescoping and PLP. This study provides insight into factors that may maintain PLP, generating targets for further investigation.

## 1 Introduction

Individuals with limb loss commonly report post-amputation phenomena, including nonpainful phantom sensations, phantom limb pain (PLP), and residual limb pain (RLP) (1–3). PLP refers to pain that is perceived in part of the limb that has been amputated, whereas RLP refers to pain that is perceived in the remaining part of the limb after amputation. Recent meta-analyses have estimated that 60%–87% of individuals who have sustained limb loss experience PLP and approximately 50%–60% experience RLP (1, 4–8). Several studies have found these two phenomena to co-occur, however, there is disagreement surrounding the relationship between post-amputation pain and nonpainful phantom sensations (6, 9, 10). This is likely due to fluctuations in several factors, including amputation etiology, post amputation complications (e.g., presence of neuromas, infection, etc.), level of amputation (e.g., upper/lower limb), and variability in research methods (e.g., survey, semi-structured interview, chart review, etc.).

One understudied nonpainful phantom sensation is known as telescoping, which is the experience of the phantom limb gradually retracting towards the residual limb and gives the impression of a shorter-than-normal phantom. Telescoping is estimated to occur in approximately one-quarter to one-third of individuals who have had an amputation (5, 11), with rates as high as 67% in persons with lower limb loss (2). Researchers have theorized that cortical reorganization is the primary mechanism underlying phantom limb telescoping (12). As the distal portion of a limb is more strongly represented at the cortical level than the proximal areas of a limb, a hand or foot may remain in perceptual awareness while other areas of the missing limb fade over time. This loss of proximal limb sensation combined with new perceptual feedback (such as a change in sensation at the level of the residual limb) may lead to cortical remapping associated with the experience of a telescoped phantom (11). It has been hypothesized that length of the phantom is a perceptual marker of the extent to which residual limb inputs have expanded into cortical areas once subserving parts of the lost limb (13, 14). Others have proposed that telescoping involves a “maladaptive” remapping process at the cortical level (12), although there are inconsistencies in research investigating the relationship between PLP and telescoping. Presently, the link between post-amputation pain and telescoping remains equivocal as it has been associated with both painful and nonpainful phantom limbs. Some studies have found telescoping is a significant predictor of lower levels of PLP in people with limb loss (14), while other studies have found the opposite (12), or no support for either relationship (2, 15). Clinicians and researchers have drawn attention to a gap in current research as it relates to better understanding the association between telescoping and PLP (16).

To the best of our knowledge, there are no descriptive reports of the course of post-amputation pain as it relates to telescoping of the phantom limb. To address this gap in research, we report the case of a research participant who experienced post-amputation pain and phantom limb telescoping with the aim of exploring the association between telescoping and ongoing PLP and RLP, in addition to other relevant psychosocial factors. We hypothesized that high levels of PLP and/or RLP would be associated with decreased telescoping, optimism, resilience, and chronic pain acceptance. Moreover, we hypothesized that greater pain levels would be associated with high pain catastrophizing, and greater symptoms of depression and anxiety.

## 2 Methods

### 2.1 Case presentation

A 35-year-old Caucasian male who underwent a traumatic transfemoral amputation was studied for the purpose of this case review. The participant provided informed consent to participate as part of a larger project (see Ethics Statement below) investigating the relationship between post-amputation pain and telescoping. On August 15, 2023, he was asked to respond to questionnaires evaluating demographic and health-related information, pain experience, and psychological factors. He also responded to questions on a computer app that evaluates telescoping experience. One month later, the participant completed a semi-structured interview, which asked him questions regarding his experiences with amputation and his phantom limb.

The participant lost his right leg in a motor vehicle collision (MVC) approximately 4.5 years prior to his participation in this study. He noted that his leg was completely severed during the collision, and he was conscious as it occurred. At the time he participated in the study, he was married and living with his wife. The participant reported that his highest level of education attainment was completing his post-secondary education. At the time of the study, he was working for a non-profit corporation in a role he held before the collision, and that he resumed approximately 2 years after the amputation. The participant reported ongoing pain problems including PLP and RLP in his right lower limb.

### 2.2 Qualitative interview

During the qualitative interview, the participant was asked a series of questions regarding his experience of PLP, RLP, and non-painful phantom sensations. The questions asked are listed in Table 1. The qualitative information was coded by two members of the research team (AA and HLR) and coded according to previously identified coding schemes (e.g., pain experience, non-painful phantom sensations, psychological impact, etc.).

**Table 1:** The list of interview questions.

No.	Questions
1.	How would you describe your phantom limb, and any pain associated with it?
a.	When have you experienced it?
b.	Has it only been in the past or also presently?
c.	When did it first start?
d.	Had it ever gone away and then came back?
e.	How would you describe the type of pain, if any?
f.	Has the feeling changed over time?
2.	How would you describe your residual limb pain, if any, which is the pain that you feel in the part of your remaining limb after your amputation?
a.	When have you experienced it?
b.	Has it only been in the past or also presently?
c.	When did it first start?
d.	Had it ever gone away and then came back?
e.	How would you describe the type of pain, if any?
f.	Has the feeling changed over time?
3.	Have you experienced phantom limb “telescoping”?
a.	When did it first happen?
b.	What were your initial reactions?
c.	How have others reacted when you have tried to explain it?
d.	What have been your experiences with it?
e.	Is it a constant length?
4.	Have you experienced any shrinkage of the phantom limb?
a.	When did it first happen?
b.	What were your initial reactions?
c.	What have been your experiences with it?
d.	Is it a constant size?
e.	How has it influenced areas of your life negatively or positively?
5.	When thinking about the different types of sensations associated with your amputation (e.g., phantom limb, telescoping, residual limb, etc.), how have any of these affected your day to day life?
a.	Impact on ability to maintain your physical health?
b.	Impact on your mental wellbeing?
c.	Impact on your relationships with others?
6.	Is there anything else about your phantom limb that you would like to discuss that we did not touch upon?

### 2.3 Questionnaires

One-month prior to the interview, the participant completed the questionnaires described below, results of which are listed in Table 2. The Brief Pain Inventory (Short Form) (BPI-SF) pain interference items were used to measure pain interference (17) across seven domains, including general activity, mood, work, sleep, and enjoyment of life. Items are scored on a scale from 0 (“does not interfere”) to 10 (“completely interferes”). The average obtained across these items has been shown to have high reliability and validity across diverse pain conditions, including phantom limb pain (18).

**Table 2:** Participant questionnaire results.

Measure	Subscale	Score	Severity/interpretation
BPI-SF	Pain interference	4.29	Mild to moderate pain interference
PCS-4	Total score	16	High pain catastrophizing
IDPQ	Total score	3	Above cut-off, high likelihood of neuropathic pain
PHQ-4	Anxiety	2	Below cut-off, mild anxiety symptoms
	Depression	2	Below cut-off, mild depression symptoms
	Total score	4	Mild anxiety and depression symptoms
LOT-R	Total score	5	Low optimism (high pessimism)
CD-RISC2	Total score	5	Below general population mean
CPAQ-8	Activity engagement	14	High
	Pain willingness	7	Low
	Total score	21	

BPI-SF, Brief Pain Inventory Short Form; PCS-4, The 4-Item Pain Catastrophizing Scale; IDPQ, Identification Pain Questionnaire; PHQ-4, The 4-Item Patient Health Questionnaire; LOT-R, Revised Life Orientation Test; CD-RISC2, The 2-Item Connor-Davidson Resilience Scale; CPAQ-8, The 8-Item Chronic Pain Acceptance Questionnaire.

The ID Pain Questionnaire (IDPQ) is a 6-item self-administered screening tool with “yes” or “no” as response options used to identify neuropathic pain (19). The six items address different characteristics of neuropathic pain, such as burning, numbness, and electrical shocks. Scores range from −1 to 5, with higher scores indicative of pain with a neuropathic component. Scores between 3 and 5 are estimated to have a 69% probability of neuropathic pain (19). The IDPQ has been demonstrated to accurately indicate the presence of a neuropathic component of pain (19).

Catastrophic thinking about pain was measured with the abbreviated measure of the Pain Catastrophizing Scale (PSC-4) (20). The PCS-4 is rated on a 5-point Likert scale from 0 (“not at all”) to 4 (“all the time”). Total scores range from 0 to 16, where higher scores are indicative of greater catastrophic thinking related to pain. The short form of the questionnaire has good internal consistency and correlates highly with scores from the original PCS (20, 21).

The 4-item Patient Health Questionnaire-4 (PHQ-4) was used as a composite measure of depression and anxiety (22). Total scores greater than or equal to 3 on the first two and last two items suggest symptoms of anxiety and depression, respectively. Overall scores range from 0 to 12 and are rated as normal (0–2), mild (3–4), moderate (6–8), and severe (9–12). The questionnaire demonstrates high reliability and validity as a measure of depression and anxiety (23). The 10-item Life Orientation Test-Revised (LOT-R) was used to evaluate dispositional optimism (24). The participant was asked to indicate the degree to which he agrees with each of the ten statements about positive and negative expectations on a scale from 0 (“strongly disagree”) to 4 (“strongly agree”). Four of the items are filler items only and do not contribute to the total score. Total scores range from 0 to 24, where a higher score is indicative of greater dispositional optimism. Scores ranging from 0 to 13 suggest low optimism (high pessimism), 14–18 suggest moderate optimism, and 19–24 suggest high optimism (low pessimism) (25). The measure contains both positively and negatively worded items which demonstrate good internal consistency and reliability across populations, including those with chronic pain (26).

The 2-item Connor-Davidson Resilience Scale (CD-RISC2) was used to measure resilience, the ability to “bounce back” and successfully adapt to change (27). Respondents are required to respond to the two items on a scale from 0 (“not at all true”) to 4 (“true nearly all the time”). Total scores range from 0 to 8, with higher scores indicative of greater resilience. Mean CD-RISC2 score in the general North American population is approximately 6.91 (27). The self-rated measure has been demonstrated to display good internal consistency, convergent validity, divergent validity, and test-retest reliability (27).

The Chronic Pain Acceptance Questionnaire (CPAQ-8) is an 8-item scale to evaluate acceptance of chronic pain (28). Respondents rate the extent to which each statement is true for them on a scale from 0 (“never true”) to 6 (“always true”). The questionnaire is comprised of pain willingness and activity engagement scales, which are combined to provide a total score ranging from 0 to 48. Higher scores are indicative of greater chronic pain acceptance. The questionnaire also has the capacity to identify four different clusters of patients with distinct functional levels (e.g., high activity engagement and pain willingness, low activity engagement and pain willingness, high activity engagement and low pain willingness, and low activity engagement and high pain willingness) (29). The CPAQ-8 has demonstrated high validity and good psychometric properties related to rehabilitation (30).

### 2.4 Telescoping app

When completing the questionnaires, the participant was also asked to respond to questions on a recent web-based/mobile application (https://demo.phantomlimbs.ca/), developed by our team to assess the extent of phantom limb telescoping and associated factors. The application allows the participant to identify the limb and level of amputation, the nature of the phantom limb, and the extent of telescoping (from normal length to completely telescoped) measured visually and as percentage (normal length phantom = 0% to completely telescoped phantom inside the residual limb = 100%) (31). This app is similar to other developped measures of phantom limb sensations (32), while requiring less time to complete and measuring other amputation-related information. The app also collects information related to participants’ age, sex, date of amputation(s), and intensity of their PLP and/or RLP on average on a numeric rating scale using a slider that moves from 0 to 10. The tool was designed as a systematic, consistent, and precise measure of telescoping that can be used to document its prevalence and understand its relationship to post-amputation pain (31).

## 3 Results

### 3.1 Qualitative interview

During the qualitative interview, the participant provided a detailed account of his phantom limb and pain experiences since the amputation. He explained that shortly after the amputation, he vividly experienced his phantom foot at the same level as his intact foot (i.e., normal length) with what he described as “intense” painful and non-painful sensations. He reported experiencing daily “extreme nerve pain” in his phantom foot for more than 1 year after his amputation. He described how it felt as though his phantom foot was “encased in cement” and painfully “compressed from all sides”. He explained that this compression feeling increased during the day, thereby increasing his pain experience. In thinking back to this period in his life, the participant rated this PLP on average as a 10 on a Numeric Rating Scale (NRS) from 0 (“no pain”) to 10 (“worst pain imaginable”). During the first year after the amputation surgery, he reported that the pain experience was a “struggle” that made him “very irritable”, and negatively impacted his relationships with others. He reported that he was not significantly impacted, emotionally or psychologically, by the loss of his leg as he was glad to have survived the MVC. Nevertheless, the PLP he experienced was “debilitating to [his] mental health”. In addition, approximately 6 months after the amputation, the participant reported that he developed a problem with the healing of the residual limb (i.e., heterotopic ossification), which delayed the use of his prosthesis and increased his discomfort until the problem was successfully treated.

The participant recalled how over time, his painful and non-painful phantom experiences “shifted”. As his pain experience changed, the participant recalled also experiencing changes to his phantom limb. He explained that approximately 1 year after the amputation, coinciding with the use of his prosthetic leg, he noticed that his phantom foot began to move up towards his residual limb. He explained that there was further shortening of the phantom limb from week-to-week, as he concurrently experienced a reduction in the intensity of his PLP.

Approximately 2 years after the amputation, he described his PLP experience as somewhat “comfortable”. At the time of the interview, approximately 4 years after the amputation, he reported his phantom foot to be attached to and partially inside the residual limb at the level of his upper thigh. The participant described how his phantom limb does not vary in length any longer, nor is it impacted by wearing his prosthetic. He described his phantom foot as the same size as the contralateral intact foot and denied experiencing any shrinkage of his phantom foot. When asked about his reaction to the telescoping experience, the participant responded: “*My biggest thing was if I just have to get over how uncomfortable phantom limbs—like pain and sensation is, then like, anything beyond discomfort, I'm going to train myself to be comfortable with. […] So, I wasn't really bothered—like, I wasn't bothered too much by having the feeling up there. It was more so just like, make it—please make it comfortable*.”

The participant explained that he now experiences a variety of different painful and non-painful phantom sensations that he can more easily cope with. While he currently continues to feel his phantom foot and ankle, he experiences “light pressure” around the foot, as if it were “under water”. He rated this frequent pressure as a 1 out of 10 on the NRS and said that it does not interfere with his daily activities. On days when he is more physically active, he reported that he will experience greater PLP intensity (increasing to 3 or 4 on 10 on the NRS), which he likened to the feeling of having an open “blister” on the lateral aspect of his phantom foot that is continuously being rubbed. He reported that this pain does not deter him from engaging in physical activity. Lastly, the participant described experiencing monthly debilitating pain which typically lasts for a period of approximately 24 h. He described the sensation as an “electric zapper” at a high intensity that is being applied to his phantom foot every 30–60 s. The participant rated this pain as 9 or 10 on the NRS and results in no sleep, “peak frustration” and “peak discomfort”. He also commented that this monthly pain is especially difficult to cope with as it often increases his feelings of frustration, which increases his pain intensity in turn. The participant explained that he started massage therapy to his residual limb approximately 1 year ago and that it “significantly helped” manage his PLP pain. Overall, he rated the average intensity of his overall pain at a 6 on the 11-point NRS. He reported that he has not experienced significant levels of RLP since his leg healed after surgery, though does occasionally experience pain when using his prosthetic leg, which he feels can be easily addressed.

### 3.2 Questionnaires

The participant scored a 3 on the IDPQ, suggesting that he experiences symptoms of neuropathic pain. His overall score on the CPAQ-8 was 21, with subscales of pain willingness and activity engagement scoring 7 and 21 respectively. He had an average score of 4.29 on the BPI-SF and a total score of 16 out of a possible 16 on the PCS-4. His total score on the PHQ-4 was 4, with scores of 2 for both the depression and anxiety subscales. He attained total scores of 5 on the LOT-R and CD-RISC2.

### 3.3 Telescoping app

**Figure 1:**
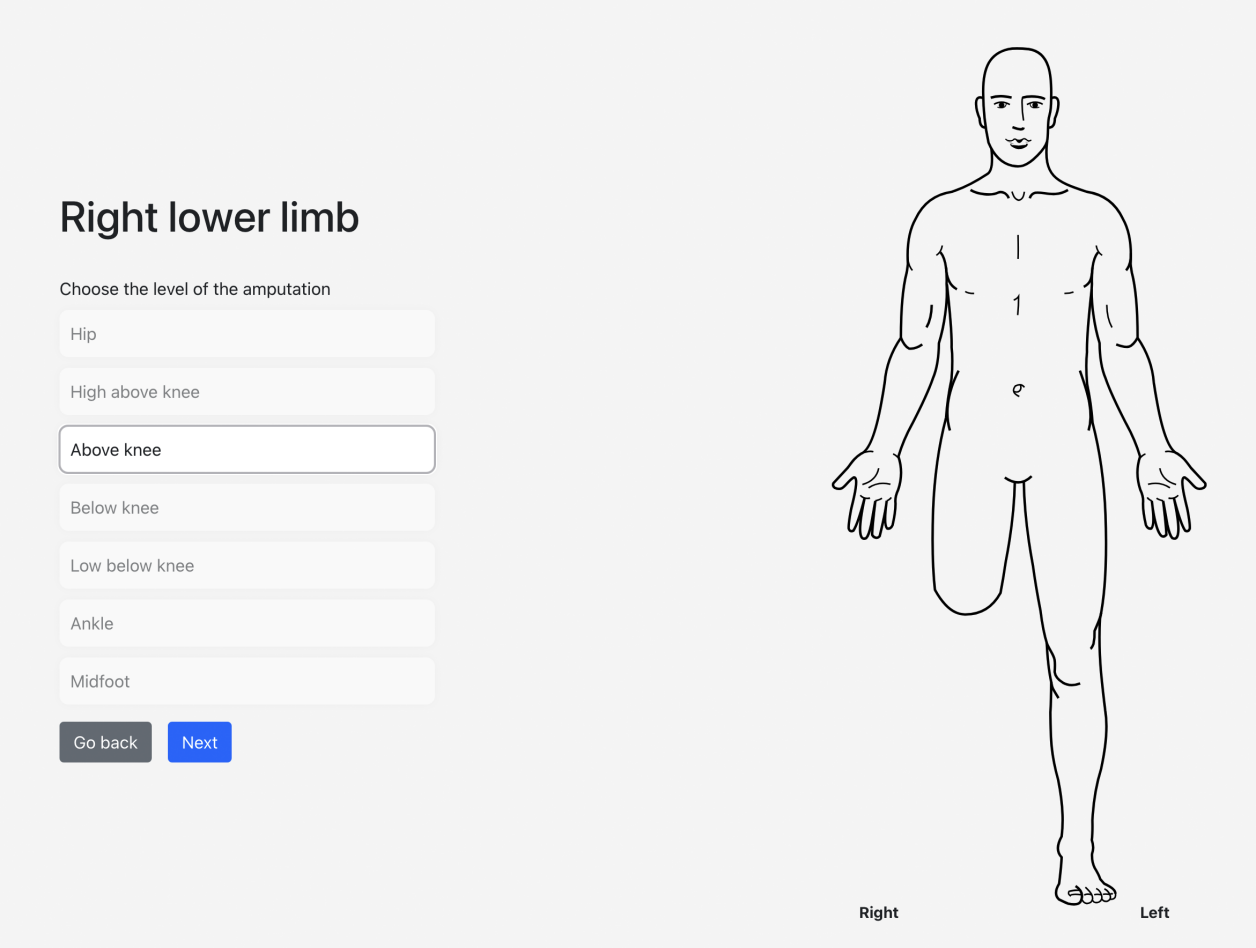
An illustration of the participant's selected level of amputation and the associated visual representation.

**Figure 2:**
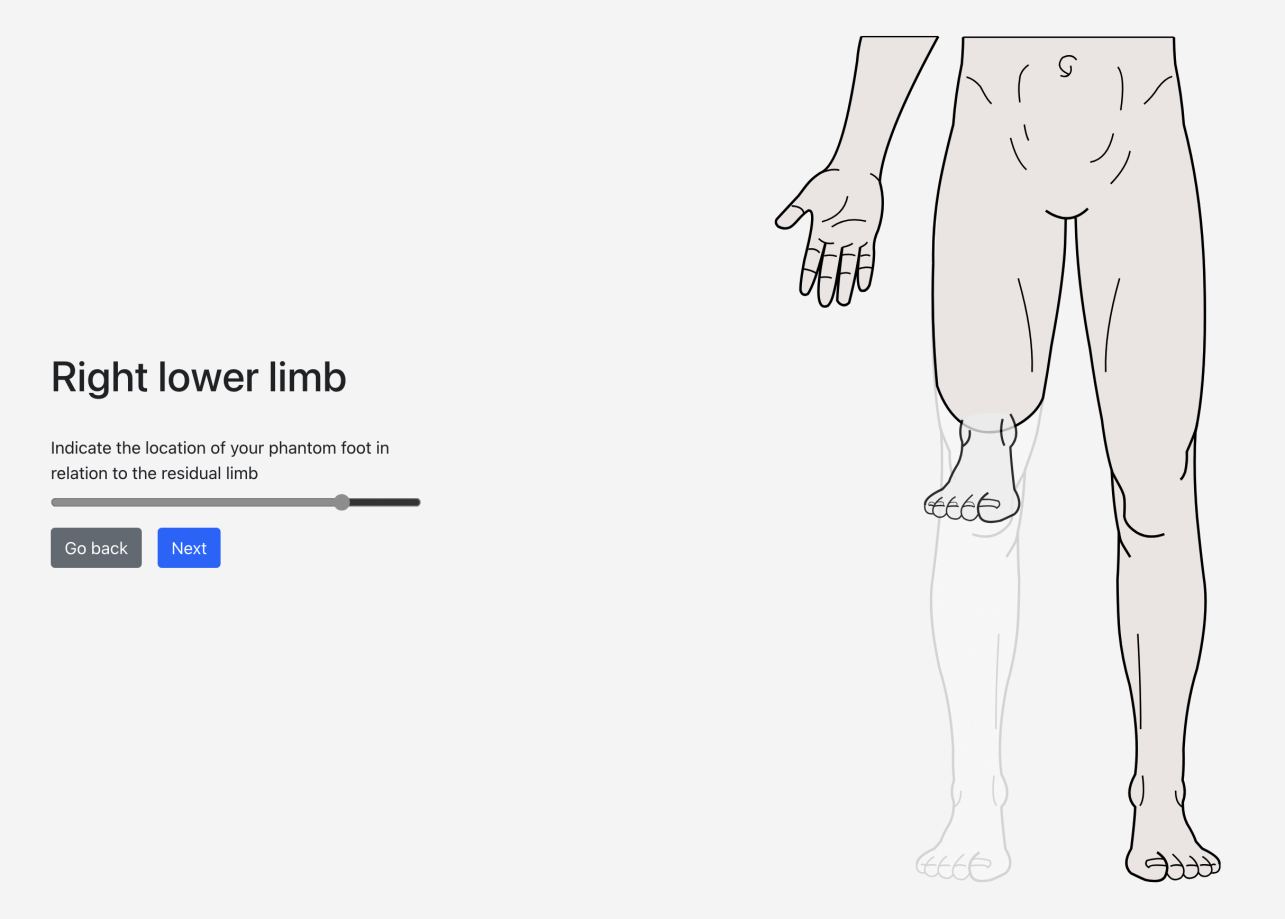
A visual representation of the participant's selected level of amputation (above knee), phantom limb (foot and ankle), and degree of telescoping (76%).

On the web-based/mobile application evaluating post-amputation pain and telescoping, the participant indicated that his phantom experience consisted mainly of his phantom foot and ankle, which were telescoped to 76% (Figures 1, 2). Using the sliders, he rated the intensity of his RLP and PLP over the past week as both 5 out of 10 on the 11-point NRS.

## 4 Discussion and future directions

This case provides information on the possible relationship between telescoping and post-amputation pain, notably PLP. To the best of our knowledge, this study is the first to thoroughly describe the onset and progression of telescoping alongside reductions in PLP and RLP intensity. The participant described in this case report experienced the onset of telescoping of his phantom limb followed by a reduction in his PLP intensity. This is consistent with our hypothesis and other studies that have found an inverse relationship between telescoping and PLP experience (13, 14). It is not possible to determine whether PLP decreased as a result of telescoping or other rehabilitation-related factors. Nevertheless, based on the present participant's experience, it is possible that telescoping is a perceptual marker of an adaptive form of plasticity reflecting a process of cortical reorganization that is accompanied by a reduction in PLP severity (13, 14). The cortical remapping that occurred as the participant's phantom foot remained in perpetual awareness and the other areas of his missing limb faded over time may have resulted in cortical reorganization that reduced his PLP.

The current case may clarify why other researchers have found PLP to be as likely to be present in a telescoped phantom as in a full-length phantom (2) and why others have considered telescoping a “maladaptive” process (12). It may not be the absolute value of the PLP intensity that is associated with telescoping but rather its reduced intensity relative to earlier levels. Thus, while telescoping and PLP can both be present, others may not have considered that a critical factor involved in telescoping is a reduction in the intensity of PLP which still could leave the individual with a significant level of PLP intensity. This possibility is best tested with a longitudinal design that observes the course of telescoping and PLP concurrently.

There are several other notable factors about this case. First, 4 years after amputation, the participant still experiences PLP, consistent with an estimated 63% of individuals after an approximate equivalent period of time (33). As well, he reported RLP at various times after amputation and at least three different qualities of PLP including a feeling of pressure as if his phantom foot were encased in cement or underwater, the sensation of a blister-like pain on his phantom foot that occurred during bouts of exercise, and monthly, intense electric-shock-like pain that lasts 24 h. This is consistent with published studies indicating that people with amputation-related pain report multiple kinds of PLP (33, 34). For example, on average, participants with PLP used a median of 13 different PLP descriptors, including shock, burning, pressure, and pins and needles (34). Moreover, PLP and RLP are strongly correlated (35–37), suggesting that many individuals with limb loss also experience a variety of RLP-related sensations, in addition to PLP. This diversity in pain experience within any given individual is partly why PLP has been so intractable with multiple mechanisms responsible for the pain (38, 39).

The finding that the participant continued to experience a shortened phantom even when wearing his prosthesis is unusual. Typically, individuals with a shortened or telescoped phantom limb report that when they are wearing a prosthesis, the phantom limb extends to normal length to match the length of the prosthetic limb as appreciated by vision, a phenomenon referred to as “embodiment” (40). Prosthesis embodiment was reported to occur significantly more often in those with a normal length phantom than in those with a telescoped phantom (40), but other cases in which the two do not coincide have been reported, including a 10-year-old boy with bilateral below-the-knee amputations performed 10 months earlier who felt his “toes at [the] stumps, not in [the] tips of [his] prosthetic shoes” (41).

The participant's responses to questionnaires suggest other factors may be contributing to his PLP. Consistent with our hypotheses, the participant endorsed moderate post-amputation pain while endorsing questionnaire items consistent with high levels of catastrophic thinking about pain, low optimism, below average resilience, and low pain willingness. Catastrophic thinking about pain has been demonstrated in several studies to be positively correlated with the severity of PLP and disability in individuals with limb loss (42–45). Contrary to our hypotheses, the participant endorsed mild symptoms of anxiety and depression and high activity engagement. Provided the participant endorsed moderate daily PLP, we would have predicted higher symptoms of anxiety and depression and lower activity engagement.

While the current case report suggests there is a link between telescoping and a reduction in PLP from a previously higher level, future large-scale studies are needed to determine whether this is in fact the case. There are several limitations to this study. Given this is a single case report, our findings likely do not reflect most limb loss patients. Additionally, all information regarding the participant's amputation experience was collected retrospectively. As a result, various biases, including recall bias, may have influenced the results. Moreover, physical and neurological examinations were not completed, limiting the information gathered from this participant. In addition, to ensure the interview was reasonable in duration, not all information about the participant's experience could be gathered. As such, the participant was not asked if he can move his phantom limb nor was he asked to list the medication he has used and if they have been helpful/unhelpful. Following the patient longitudinally through the various stages of his rehabilitation would have been advantageous. The inclusion of a variety of different individuals with limb loss in future studies will be necessary to confirm the validity of a possible association between telescoping and PLP.

Using the valuable insights offered by this case report, we are exploring the use of targeted, customizable interventions, such as virtual reality (VR), to further evaluate the relationship between telescoping and PLP. Immersive VR environments can simulate telescoping experiences, measure associated changes in PLP intensity and frequency, and have demonstrated efficacy in pain management (46). Such an innovative approach holds potential for uncovering and better understanding the underlying mechanisms linking telescoping and PLP (47). Through rigorous evaluation in clinical settings, the efficacy of this novel VR tool can be assessed, offering a promising avenue for addressing the complex interplay between post-amputation phenomena and improving the quality of life for individuals with limb loss (48).

## 5 Conclusions

This case report describes the experiences of a person with a lower limb amputation focusing mainly on PLP and phantom limb telescoping. The participant described the onset of telescoping followed shortly by a reduction in PLP intensity. Future adequately powered longitudinal studies are needed to fully explore this relationship. In addition, mechanism-based interventions are needed to determine the temporal and potential causal relationships between the two. It is hoped that this case report will contribute to better understanding the factors that influence post-amputation experience and rehabilitation for individuals with limb loss.

## Data Availability

The raw data supporting the conclusions of this article will be made available by the authors, without undue reservation.

## Participant perspective

The participant read this case report and agreed with the content. He provided written informed consent to have the case report published.
